# Particle Beam Therapy for Cancer of the Skull Base, Nasal Cavity, and Paranasal Sinus

**DOI:** 10.5402/2012/965204

**Published:** 2012-05-31

**Authors:** Nobuyoshi Fukumitsu

**Affiliations:** Department of Radiation Oncology, Ibaraki Prefectural Central Hospital, 6528, Koibuchi, Kasama 309-1793, Japan

## Abstract

Particle beam therapy has been rapidly developed in these several decades. Proton and carbon ion beams are most frequently used in particle beam therapy. Proton and carbon ion beam radiotherapy have physical and biological advantage to the conventional photon radiotherapy. Cancers of the skull base, nasal cavity, and paranasal sinus are rare; however these diseases can receive the benefits of particle beam radiotherapy. This paper describes the clinical review of the cancer of the skull base, nasal cavity, and paranasal sinus treated with proton and carbon ion beams, adding some information of feature and future direction of proton and carbon ion beam radiotherapy.

## 1. Introduction

Particle beam therapy was first proposed in the 1940s [1] and was investigated in the USA, Sweden, and the Soviet Union in the 1950–1960s. In the 1970s, particle beam therapy was rapidly developed in parallel with the development of X-ray computed tomography. Globally, more than 48,000 patients have been treated with particle beams. Most of these treatments were delivered using proton and carbon ion radiotherapy (RT). Currently, there are 36 particle beam therapy facilities in operation (proton: 33, carbon ion: 5, both particles: 2), according to the Particle Therapy Co-Operative Group homepage. Ten facilities are located in the USA, followed by eight in Japan, three in Germany, three in Russia, two in France, and 10 in other countries (Table 1).

This paper describes the status and prospects of particle beam therapy for the treatment of cancer of the skull base, nasal cavity, and paranasal sinus, including physical, biological, technical, and financial aspects.

## 2. Physical, Biological, Technical, and Financial Aspects of Particle Beam Therapy

The physical, biological, technical, and financial aspects of particle beam therapy differ largely from those of conventional photon RT.

### 2.1. Physical Aspects

**Figure 1:**
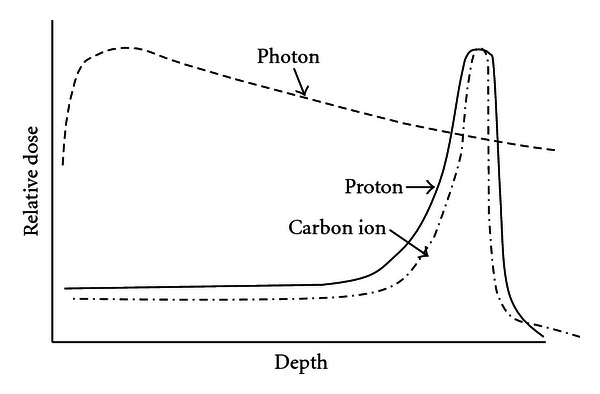
Relationship of depth and relative dose in photon, proton and carbon ion beams. Proton and carbon ion beams can produce Bragg peak. The particle range is determined by the energy and larger radiation dose can be delivered to the tumor than photon beam.

Both proton and carbon ion beams have features that are extremely different from those of photons. Accelerated proton and carbon ion beams show an increase in energy deposition with penetration depth up to a sharp maximum followed by rapid decrease at the end of the penetration range; this phenomenon is known as the Bragg peak (Figure 1). The particle range is determined by the energy of the incoming particles, and the Bragg peak can be spread out. These features permit a more precise and conformal dose localization to the target, compared with photon beams.

### 2.2. Biological Aspects

Proton and carbon ion beams lose energy when they penetrate a material. With an increasing atomic mass and electric charge of the ions, the ionization density or linear energy transfer (LET) increases. Proton beams have a higher LET than photons; however, their radiobiological properties do not differ substantially from those of photons. For clinical applications, the absorbed dose is multiplied by a factor of 1.1 to express the biological effective dose in cobalt gray equivalents (GyE). Carbon ion beams have a biological advantage over photon and proton beams. Their biological efficiency increases at the end of the beam's range, whereas it is low along the entrance channel. The local values for relative biological effectiveness (RBE) are approximately 3 for carbon ion RT, but depend on many factors. Because carbon ion beams are high-LET ion beams, they can create clusters of DNA damage that cannot be repaired, they are effective for the treatment of hypoxic cells, they cause fewer variations in radiosensitivity related to the cell cycle, and fractionated irradiation is less likely to lead to the repair of radiation-induced injury.

### 2.3. Technical Aspects

Rotating gantries for proton therapy have been installed in more than half of all proton therapy facilities, especially those facilities that have newly opened in the 21st century. Although carbon ion beams have approximately three times the magnetic rigidity of proton beams and are difficult to deflect, they are still delivered in a fixed manner using horizontal or vertical beam lines. The particle therapy facility at the Heidelberger Ionenstrahl-Therapie Centre, Heidelberg, Germany (HIT) has investigated the use of a rotating gantry for carbon ion beams, but this setup has not yet been realized.

**Figure 2:**
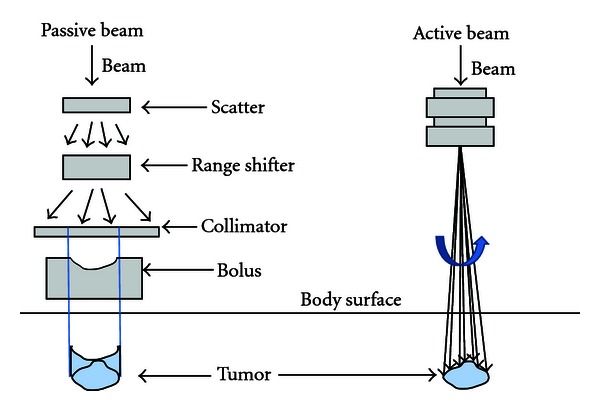
Schema of passive (left) and active (right) particle beams (the systems vary somewhat among institutions).

Beam delivery has been performed using passive methods with modulators, collimators, and compensators at most facilities. The beam is bended using electromagnets and spread by scattering. A ridge filter alters the width of the Bragg peak, and a range shifter adjusts the beam range. The collimator and bolus regulate the outline and depth of the beam based on the target (the systems vary somewhat among institutions). The advantage of a passive beam delivery system is that the treatment planning for this system is simple and can easily produce a conformal irradiation dose, both laterally and at the correct depth, relative to the target. On the other hand, the main disadvantage is that the area along the entrance path is also unavoidably irradiated, and this area often includes nontarget normal tissue. As an alternative to passive beam delivery, active beam delivery techniques, such as spot scanning or raster scanning, have been developed using narrow pencil beams [2, 3]. Pencil beams produced in a synchrotron are deflected laterally by two magnetic dipoles, whereas the energy of the incoming beam is varied during the treatment. Thus, a three-dimensional, single-spot Bragg peak is created that conforms to the target after magnetic deflection, and the dose distribution can be tailored optimally to any irregular tumor shape. The dose distribution can also be conformed to the proximal edge of the target volume, and normal tissue that resides along the entrance channel of the beam can thus be spared (Figure 2).

### 2.4. Financial Aspects

Although particle beam therapy has great physical and biological advantages, only a few facilities throughout the world can perform this treatment, as mentioned above. One of the most important disadvantages of particle beam therapy is the high cost of its technical realization and operation. Large cyclotrons or synchrotrons are needed to accelerate protons and carbon ions to the required energy levels for the treatment of deep-seated tumors. A cost analysis study in Europe calculated the capital costs of proton beam RT to be approximately €60–80 million and that of carbon ion beam RT to be €160 million [4]. The operation costs are €16–18 million per year for both proton and carbon ion beam RT. Furthermore, extremely precise and reproducible patient positioning coupled with high-quality imaging for treatment planning is prerequisites for this type of treatment.

## 3. Skull Base Tumors

Chordoma and chondrosarcoma are the most common skull base tumors to be treated using particle beam therapy [5–16] (Table 2). Chordoma and chondrosarcoma are characterized by a high histologic differentiation, locally aggressive growth, and high recurrence rates. Surgery is the first choice for the treatment of skull base tumors. However, complete resection is difficult and challenging because of the proximity to vulnerable critical organs, such as the optic nerve and brainstem. These anatomic structures often limit surgical resection, and residual tumor tissue results in a high recurrence rate. In addition, many patients are reluctant to undergo gross surgery for cosmetic reasons. Metastasis to lymph nodes in the neck is uncommon for these tumors. Hence, prophylactic neck irradiation is not necessary, and an involved field technique is usually used for RT. Gadolinium contrast magnetic resonance imaging (MRI) offers better soft tissue contrast, which is the most important feature in radiation treatment planning, and diffusion-weight MRI or fluorine-18 deoxyglucose (^18^FDG) positron emission tomography imaging can provide important information regarding tumor delineation and activity. The investigation of focal dose escalation to substantially inhomogeneous tumors using ^18^FDG or other tracers, such as fluorine-18 fluoromisonidazole, that are suited for the detection of hypoxic cells has been performed for head and neck cancers; however, useful clinical evidence has not yet been obtained.

Munzenrider reported the long-term follow-up data for 519 patients with chordoma or low-grade chondrosarcoma who were treated with proton beams and photons [6]. The patients received 66–83 GyE, and the overall survival rate (OS) at 10 years was 54% for the patients with chordomas and 88% for the patients with chondrosarcomas. Myelopathy, neuropathy, hearing loss, and endocrinopathy were severe side effects. Colli reported that chordoma patients treated with proton beams with or without photons had a significantly higher recurrence-free survival rate than patients treated with photons in 2001 [7]. Many clinical studies have been performed using proton and carbon ion beams for the treatment of skull base tumors during this past decade. HIT has traditionally reported updated data for skull base tumors [9, 11, 14, 17]. Schulz-Ertner reported that the three-year OS was 91% and the local control rate (LC) was 81% in chordoma patients and that the three-year OS was 98% and the LC was 96% in chondrosarcoma patients; severe late side effects were observed in less than 2% of the patients in their study of more than 50 patients using carbon ion beams [9, 14]. Combs reported data for young adult patients with chordoma or chondrosarcoma who were treated with carbon ion beams [16]. A total of 18 patients (5–21 years old) were treated with carbon ion beams of 60–66.6 GyE; the OS was 100%, and the local control rate was 94% in the 3–112-month follow-up period. Severe late side effects were not found. A report of further long-term follow-up data is expected. In total, many patients received a gross or partial resection before radiotherapy, and the irradiation dose was approximately 60–70 GyE and the OS was 80–90% at 3–5 years after treatment. Age, tumor volume, extension, surgical history, and visual function at presentation were pointed out as prognostic factors in some reports. Late side effects were rare in all the studies. Pommier et al. reported that 10 out of 23 adenoid cystic carcinoma (ACC) patients who received 70–79.1 GyE of proton beams and photons exhibited a grade 3 neurologic deficiency (seizures in 7 patients and short-term memory loss in 3 patients); however, all the seizures were controlled with medication [12].

In summary, the effectiveness of conventional photon RT is limited in patients with chordomas and chondrosarcomas, and postoperative high-dose proton or carbon ion beam RT exhibits excellent tumor control without late side effects and should be selected as an adequate treatment of choice.

In recent years, the Paul Scherrer Institute (PSI) and HIT have started active proton and carbon ion beam radiotherapy for the treatment of chordoma and chondrosarcoma patients. Active beam particle beam therapy is, theoretically, extremely adequate for the treatment of skull base tumors in which the tumor and critical organs, such as the optic nerve and brainstem, are tangled together. HIT has also started a prospective comparative phase study of proton and carbon ion beams for the treatment of chordoma and low- and intermediate-grade chondrosarcoma [18, 19]. The results of these studies will be published in the near future.

## 4. Nasal Cavity and Paranasal Sinus Cancers

Squamous cell carcinoma (SCC), malignant melanoma (MM), and ACC are the most common nasal cavity and paranasal sinus cancers to be treated using particle beam therapy [13, 20–25] (Table 3). Surgery or radiotherapy is the treatment of first choice for nasal cavity and paranasal sinus cancers, and combined treatment is recommended for locally advanced cancers. Complete resection is difficult and challenging for the same reason as for skull base tumors, and the use of high-dose curative radiotherapy with or without chemotherapy is increasing. These anatomic structures often limit surgical resection, and residual tumor tissue results in a high recurrence rate. The necessity of prophylactic neck irradiation is controversial because of the variations in tumor histology, site, and volume. However, an involved field technique is frequently used for locally advanced N0 cancers. Gadolinium contrast MRI, diffusion-weight MRI, and ^18^FDG are useful for treatment planning.

The National Institute of Radiological Sciences, Chiba, Japan (NIRS) reported a dose escalation study examining carbon ion beam RT for head and neck cancer patients [20]. Because this study included many kinds of head and neck cancer patients, the outcome of only nasal cavity and paranasal sinus cancer patients is difficult to extract. However, this study revealed that carbon ion RT consisting of 70.2 GyE administered in 18 fractions over 6 weeks and carbon ion RT consisting of 64 GyE administered in 16 fractions over 4 weeks had equal clinical outcomes in terms of morbidity and local control. In addition, the treatment effectiveness differed largely according to tumor histology, and MM had a better LC than SCC.

Previous studies of nasal cavity and paranasal sinus cancer can be divided into two types according to tumor histology: mainly SCC and non-SCC. In a study examining mainly SCC, the irradiation dose was approximately 60–80 GyE and the OS was 50–90% at 2-3 years after treatment. The OS was 90% and the disease-free survival rate was 77% at 3 years in a study in which 78% of the patients, who had mainly SCC, received a gross resection before undergoing RT using proton beams and photons (60.8–77 GyE) [21], whereas the OS was 59% at 3 years and 47% at 2 years in studies in which no patients received any kind of resection before undergoing RT [23, 25]. In studies examining non-SCC, the irradiation dose was approximately 50–80 GyE and the OS was 50–80% at 3 years after treatment. Jingu at the NIRS reported an OS of 81% and an LC of 65% among MM patients who were treated with 57.6 GyE of carbon ion beams with concurrent chemotherapy. The tumor volume, site, and extension were identified as prognostic factors in some reports. Overall, reported severe late side effects have included brain necrosis, bone necrosis, and deteriorations in visual function; however, either the morbidity rate was quite low or the morbidities were inevitable and the patients' consensus had been obtained in each of the studies.

In summary, the OS of patients with nasal cavity or paranasal sinus cancer varies widely. The rarity of such tumors makes it difficult to perform large studies and may cause a patient bias at each institute. Consequently, comparisons among studies are also difficult. Further studies involving larger numbers of patients are needed.

## 5. Discussion and Future Directions

Particle therapy with protons and carbon ion beams offers physical and biological advantages, compared with conventional photon RT. On the other hand, a complex technique is needed, and the capital and operation costs are relatively high. Particle beam therapy is advantageous for the treatment of tumors such as skull base, nasal cavity, and paranasal sinus cancers, as these tumors are often located close to critical organs. A clinical review has revealed that high-dose irradiation is a feasible and safe treatment. To further improve the applicability of particle beam therapy, future developments in the fields of imaging and radiation technology must be fully integrated into the particle therapy process. For example, most modern treatment planning systems include advanced image registration functions. As imaging technologies improve, intensive irradiation dose planning will become more accurate, and an active beam delivery technique will be able to deliver a focally intensive radiation dose to the tumor while avoiding critical organs in the vicinity. Regarding radiation technology, a rotating gantry of carbon ion beams, which is presently under investigation, will be able to overcome the disadvantage of carbon ion beams and should be capable of excellent treatment effects once it is realized.

Some clinical studies have examined the treatment of skull base, nasal cavity, and paranasal sinus cancer patients using particle beam therapy. However, because of the rarity of such tumors, definite conclusions were not obtained. Prospective trials involving a large number of patients are needed to obtain clinical evidence. Moreover, past studies have mainly focused on OS, LC, and side effects. In recent years, photon RT is also technically progressing especially development of intensive modulated radiotherapy (IMRT). To apply particle beam therapy widely, a cost performance analysis comparing conventional RT or IM RT is very important in the future studies.

## Figures and Tables

**Table 1:** Institution of particle beam therapy.

Institution	Country	Particle	Start of treatment
ITEP, Moscow	Russia	P	1969
St. Petersburg	Russia	P	1975
PSI, Villigen	Switzerland	P	1996
Dubna	Russia	P	1999
Uppsala	Sweden	P	1989
Clatterbridge	England	P	1989
Loma Linda	CA, USA	P	1990
Nice	France	P	1991
Orsay	France	P	1991
iThemba Labs	South Africa	P	1993
IU Health PTC, Bloomington	IN, USA	P	2004
UCSF	CA, USA	P	1994
HIMAC, Chiba	Japan	C-ion	1994
TRIUMF, Vancouver	Canada	P	1995
HZB (HMI), Berlin	Germany	P	1998
NCC, Kashiwa	Japan	P	1998
HIBMC, Hyogo	Japan	P	2001
HIBMC, Hyogo	Japan	C-ion	2002
PMRC (2), Tsukuba	Japan	P	2001
NPTC, MGH Boston	MA, USA	P	2001
INFN-LNS, Catania	Italy	P	2002
Shizuoka Cancer Center	Japan	P	2003
Southern Tohoku PTC, Fukushima	Japan	P	2008
WPTC, Zibo	China	P	2004
MD Anderson Cancer Center, Houston	TX, USA	P	2006
UFPTI, Jacksonville	FL, USA	P	2006
NCC, IIsan	South Korea	P	2007
RPTC, Munich	Germany	P	2009
ProCure PTC, Oklahoma City	OK, USA	P	2009
HIT, Heidelberg	Germany	P	2009
HIT, Heidelberg	Germany	C-ion	2009
UPenn, Philadelphia	PA, USA	P	2010
GHMC, Gunma	Japan	C-ion	2010
IMPCAS, Lanzhou	China	C-ion	2006
CDH Proton Center, Warrenville	IL, USA	P	2010
HUPTI, Hampton	VA, USA	P	2010
IFJ PAN, Krakow	Poland	P	2011
Medipolis Medical Research Institute, Ibusuki	Japan	P	2011

P: proton, C-ion: carbon ion.

**Table 2:** Particle beam therapy: skull base tumor.

Author, year	Institution	*N *	Disease	Conc. TX	Beam	Dose (GyE)	OS
Munzenrider and Liebsch, 1999 [6]	MGH	519	Chord (56%)	Unknown	P + ph	66–83	Chord: 54% (10 Y)
			Chondro (44%)				Chondro: 88% (10 Y)

Nöel et al., 2003 [8]	CPO	67	Chord (70%)	Res (91%)	P + ph	60–70 (67)	Chord: 88% (4 Y)
			Chondro (30%)				Chondro: 75% (4 Y)

Schulz-Ertner et al., 2004 [9]	GSI	54	Chord	Res (100%)	C-ion	prim: 60 or 70 (60)	91% (3 Y)
						rec: 45–60 (51)	

Igaki et al., 2004 [10]	Tsukuba	13	Chord	Res (53%)	P + (ph)	69–105 (77)	67% (5 Y)

Schulz-Ertner et al., 2005 [11]	GSI	29	ACC	Res (69%)	C + ph	72	76% (4 Y)

Pommier et al., 2006 [12]	MGH	23	ACC	Unknown	P + ph	70–79.1 (76.4)	77% (5 Y)

Tsujii et al., 2007 [13]	NIRS	25	Chord	Res (100%)	C-ion	48–60.8	86% (5 Y)

Schulz-Ertner et al., 2007 [14]	GSI	54	Chondro	Res (100%)	C-ion	57–60 (60)	98.2% (4 Y)

Ares et al., 2009 [15]	PSI	64	Chord (66%)	Unknown*	P	Chord: 67–74 (73.5)	Chord: 62% (5 Y)
			Chondro (34%)			Chondro: 63–74 (68.4)	Chondro: 91% (5 Y)

Combs et al., 2009 [16]	HIT	18	Chondro (59%)	Res (52%)	C-ion	60–66.6 (60)	100%
			Chord (41%)				

Abbreviation; *N*: number, OS: overall survival rate, GyE: Gray equivalent.

Chord: Chordoma, Chondro: Chondrosarcoma, ACC: Adenoid cystic carcinoma, Res: gross or partial resection, P: proton, ph: photon, C-ion: carbon ion, prim: primary, rec: recurrence.

*: Res (100%) including biopsy.

**Table 3:** Particle beam therapy: nasal cavity and paranasal sinus cancer.

Author, year	Institution	*N *	Disease	Conc. TX	Beam	Dose (GyE)	OS
Weber et al., 2006 [21]	MGH	36	SCC (28%)	Res (78%)	P + ph	60.8–77 (69.6)	90% (3 Y)
			ACC (28%)				

Tsujii et al., 2007 [13]	NIRS	224*	MM (36%)		C-ion	57.6	57% (3 Y)
			ACC (29%)				

Tamtruong et al., 2009 [22]	MGH	20	SCC (50%)	Res (35%)	P + ph	66–78 (76)	53% (2 Y)
			ACC (35%)	Chemo (30%)			

Zenda et al., 2010 [23]	NCC	39	SCC (28%)	Chemo (3%)	P	60–70 (65)	59% (3 Y)
			ONB (23%)				

Jingu et al., 2011 [24]	NIRS	37	MM (100%)	Chemo (100%)	C-ion	57.6	81% (3 Y)

Fukumitsu et al., 2011 [25]	Tsukuba	17	SCC (65%)	Chemo (12%)	P + (ph)	72.4–89.6 (78)	47% (2 Y)
			AC (12%)				

*N*: number, OS: overall survival rate, Conc. TX: concurrent treatment, GyE: Gray equivalent.

SCC: squamous cell carcinoma, ACC: adenoid cystic carcinoma, MM: malignant melanoma, ONB: olfactory neuroblastoma, AC: adenocarcinoma, Res: gross or partial resection, Chemo: chemotherapy, P: proton, ph: photon, C-ion: carbon ion. *Including skull base tumor.
